# Visual reflexive attention as a useful measure of development

**DOI:** 10.3389/fpsyg.2023.1206045

**Published:** 2023-08-23

**Authors:** Rebecca A. Lundwall

**Affiliations:** Psychology Department, Brigham Young University, Provo, UT, United States

**Keywords:** reflexive attention, infancy, childhood, development, exogenous attention

## Abstract

Cognitive psychology began over three-quarters of a century ago and we have learned a great deal in that time, including concerning the development of cognitive abilities such as perception, attention, and memory, all of which develop across infancy and childhood. Attention is one aspect of cognition that is vital to success in a variety of life activities and, arguably, the foundation of memory, learning, problem solving, decision making, and other cognitive activities. The cognitive abilities of later childhood and adulthood generally appear to depend on the reflexes, abilities, and skills of infancy. Research in developmental cognitive science can help us understand adult cognition and know when to intervene when cognitive function is at risk. This area of research can be challenging because, even in typical development, the course of cognitive development for a particular child does not always improve monotonically. In addition, the typical trajectory of this development has been understood differently from different historical perspectives. Neither the history of thought that has led to our current understanding of attention (including its various types) nor the importance of developmental aspects of attention are frequently covered in training early career researchers, especially those whose primary area of research in not attention. My goal is to provide a review that will be useful especially to those new to research in the subfield of attention. Sustained attention in adults and children has been well-studied, but a review of the history of thought on the development of reflexive attention with a focus on infancy is overdue. Therefore, I draw primarily on historical and modern literature and clarify confusing terminology as it has been used over time. I conclude with examples of how cognitive development research can contribute to scientific and applied progress.

## Introduction

The cognitive ability to select, consciously or unconsciously, what we will attend to is foundational to many cognitive activities. It is often understood from an information processing approach (Mandler, 2002). The need to select only some items from a vast array of possible targets implies capacity limits. Because we are not able to process all the information in our environments, we must (willfully) or allow our brains to (automatically) select which information to attend to. Broadbent (1958) developed these ideas into the filter model of attention. He described attention as the process of selecting some information and blocking other information. Treisman (1969) later refined the description of filtering processes as attenuating irrelevant messages rather than blocking them.

Another way of saying that we select information, is to say that we attend to something. Attention is a foundation for memory, learning, problem solving, decision making, and many other cognitive activities (Johnson et al., 1991; Huijbregts et al., 2002; Chun and Turk-Browne, 2007; Moore and Zirnsak, 2017). Researchers have talked about the biological significance of automatic selection of information (reflexive orienting) for survival (e.g., becoming aware of dangers) but also about attention’s importance to reading (Willows, 1974), memory for things attended to (Baddeley and Hitch, 1974), curiosity (Pavlov, 1927) and perceiving emotion and intention so that we can engage socially with one another (Tomasello et al., 2005; Herrman et al., 2007; Freiwald, 2020). Attention is hugely important, but it is also complex. Therefore, this review will cover one type of attention: reflexive attention.

Reflexive attention occurs when something in our external environment grabs our attention. It is the automatic orienting of the brain’s resources toward the new object or location such as following the appearance or movement of a stimulus. Reflexive attention is useful in identifying threats or other urgent situations that were not our focus or even in our awareness (Hopfinger and Mangun, 1998; Smith and Chatterjee, 2008). Reflexive attention influences multiple stages of information processing (Öhman, 1979; Hopfinger and Mangun, 2001) and is also influenced by our experiences, such as when we develop a sensitivity to hearing our name (Cherry, 1953; Newman, 2005; Nakane et al., 2016; Röer and Cowan, 2021). Reflexive attention is an ability that can be measured in infancy, later childhood, and adulthood. However, reflexive attention has not been discussed and studied in the same ways over the course of history. The fact that attention involves and is involved with several different cognitive processes (Oakes and Rakison, 2020, pp. 60–91) can also lead to some confusion. This can make what we know about reflexive attention unclear and its usefulness obscure to researchers new to studying attention.

In this article, I start with a section on the typology and the terminology of attention. Next, I review the study of reflexive attention through history. Then I will discuss how reflexive attention is typically measured in adults and children as a foundation for my discussion of measuring reflexive attention in infants. This last section on infancy goes into some depth, including techniques and findings unique to infancy. I conclude with the predictive value of measuring infant reflexive attention for childhood cognitive functioning.

## Typology and terminology

When people think of attention, they often think of effortful attention, also called sustained attention. Sustained attention represents vigilance or concentration because of a person’s goals. It is important and allows us to concentrate on a task. Reflexive attention, on the other hand, is the automatic orienting of the brain’s resources to a new object or location due to a change in the external environment. To illustrate why we might have evolved two attention systems, consider a prehistoric woman hunting a squirrel for dinner. Meanwhile, unbeknownst to our hunter, she is being hunted by a saber-toothed tiger. If she has better sustained attention than reflexive attention, she will focus on the squirrel and not notice the saber-toothed tiger. If she is not distractible at all (has poor reflexive attention), that means she will likely be dinner before she obtains dinner. She needs both to obtain food and notice threats in her environment. Threats are quite different today. However, consider the need for reflexive attention when crossing the street, interacting with threatening people, and on vacation in unfamiliar wildernesses. We still need reflexive attention. In fact, we use it unwittingly and nearly constantly in our everyday lives.

Several researchers (Maltzman, 1979; Öhman, 1979; Johnston et al., 1995; Hopfinger and Mangun, 2001) agree that there are two attentional processes. Research evidence also exists for separate mental processes for reflexive and sustained attention (Schneider, 1969). According to this conception, orienting is a prerequisite for learning because information can only be stored in long-term memory if it has been oriented to and receives further processing by attention and working memory. As Neville et al. (2013) has said, considerable evidence supports selective attention as important in all aspects of learning, including memory and school readiness (Blair and Razza, 2007; Duncan et al., 2007; Stevens and Bavelier, 2012).

Terms I have used already, such as orienting, can be confusing. “Orienting” is imprecise because it can refer to volitional orienting when the person chooses to change focus, although reflexive attention is often meant. We can thank Maltzman (1979) for the suggestion to distinguish voluntary from involuntary orienting. Another unclear term is “selective attention.” It is less precise than necessary for our current understanding of attention and cognitive processes. A search for selective attention in academic databases returns studies using both reflexive and sustained attention tasks. Because items in attention can be selected for attention in two ways (automatically or effortfully), we need different terminology to distinguish automatic attentional processes from volitional processes.

Reflexive attention has also been described as a “preattentive process” to indicate parallel information processing that reduces the load on more complete serial-processing mechanisms (Neisser, 1967). What receives preattentive processing may depend not only on novelty but on biological needs, previous learning, or instructions. Reflecting on these contributions, you may recall that Neisser eventually became disillusioned with information processing approaches and turned to more ecological Gibsonian concepts (Neisser, 2007) and Maltzman, who also studied orienting, referred to himself as “an old unregenerate behaviorist” (Donchin et al., 1984, p. 45). Obviously, there are other approaches to understanding reflexive attention than information processing approaches. Some of these will be discussed in the next section.

In this article, I will typically use the term reflexive attention (aka exogenous attention) to discuss automatic orienting to a stimulus. I will use “sustained attention” (aka endogenous attention) to discuss attention that requires effort. However, I will use the terms of the authors whose contributions I discuss when necessary to convey their intended meaning.

## History of reflexive attention

### Early historical foundations in physiology

The study of reflexive attention has an interesting history that shows the influences of various theoretical perspectives leading to the most common information processing approach used today. In this section I will discuss the influence of psychophysiological (reflex-based), behavioral, Gestalt, and information processing approaches on studies of reflexive attention.

Although the Greek physician Galenus, 127–200 AD, also described somatic reflexes (see Hodge, 1890), the early modern emphasis on reflexes can be traced to the eighteenth century in what Boring (1950) calls the Western European “beginnings of psychophysiology” (pp. 27–49.). As an example of these beginnings, consider Robert Whytt (1751), who described involuntary, or reflexive, movements as “spontaneous” and “automatic” with “no time for the exercise of reason” (p. 312). Hundreds of works on reflexes followed, and almost a century later Johannes Peter Müller (1837) synthesized the research in his textbook, *Elements of Physiology*. In some ways, his textbook foretokened the information processing approaches in psychology. For example, Müller repeatedly wrote of attention modulating the intensity of what we perceive. In particular, he wrote, “Among a certain number of simultaneous sensations we are able to direct our attention to a single one, so as to perceive it, not only more distinctly than the rest, but definedly and in its whole intensity” (p. 622). The textbook is widely respected for its scientific rigor and was the most prominent textbook in physiology for most of the 1800s (Simonsz, 2010). Other influential researchers of this age are discussed under the information processing approach at the end of this section.

The first systematic effort to base the workings of the human mind on the physiology of reflexes stems from Sechenov’s nineteenth-century classic *Reflexes of the Brain* (1863 as translated). He believed that reflex structures underlie all psychology. The text contains ideas that were later empirically confirmed by Sherrington (1906). Sechenov is commonly considered the father of Russian physiology and, like the Russians Pavlov and Sokolov, he belonged to a group of scientists who conducted much of the research on reflexes (Ushakova, 1997).

Sokolov's (1960) ideas were somewhere between an information processing model of cognition and psychophysiology. For example, he believed that an incoming stimulus was compared to a neuronal model stored in the brain. Sokolov (1966) later added to this comparator model that the brain makes several predictions, each with its own probability. The model implies that there is a memory trace for previous stimuli and these familiar stimuli will not induce as large a neural response as novel stimuli. Some researchers have found support for the comparator model. For example, Clifford and Williston (1993) used two oddball tasks: one passive with no task requirements, for which participants were told there would be no questions after the task, followed by an active task, in which participants were asked to count rare oddball events. The study tested Öhman's (1979) proposal regarding Sokolov’s (1960) model that orienting occurs when either a stimulus cannot be matched to a representation in the brain, or it matches stimulus flagged as relevant. While there were some differences in results between the tasks (e.g., the active task elicited additional posterior-superior brain activity), the authors took the essentially equivalent event-related potential (ERP) amplitudes at 100 msec after an oddball event as evidence that stimulus detection is occurring in both passive and active tasks and comparator model orienting is evidenced by the posterior-superior activity in the active task.

Nevertheless, several other studies do not support the comparator model. For example, Richmond et al. (2007) used visual paired-comparison and novelty preference tasks and found that delays of 3 minutes, 24 hours, 1 week, 2 weeks, 6 months, and 12 months led to results that could not be explained by a simple comparator model. Instead, the results suggest that the accessibility of the representation determined whether there was orienting. Furthermore, Mackintosh (1987) argues that there is no good evidence to support Wagner’s (1976) version of the comparator model of habituation (although Wagner’s model is quite specific and perhaps easier to refute). Macintosh says that Wagner’s findings can be explained by stimulus–response theory, in which “every presentation of a stimulus causes some decline in the efficiency of transmission along an [stimulus–response] pathway” (p. 94). Mackintosh believes that calling recognition of a stimulus a comparison adds relatively little to theoretical prediction.

While there is less evidence to support the comparator model, Sokolov’s work led to more interest in studying the orienting response. Eventually, with the development of ERP, the study of reflexive orienting (i.e., reflexive attention) became more concrete. Sutton et al. (1965) made advancement with the discovery of the positive ERP component at about 300 msec. This supported Sokolov’s idea that reflexive attention is, in fact, reflexive (Voronin and Sokolov, 1960). Eventually, Näätänen and Michie (1979) described the close relationship between mismatch negativity and the orienting reflex, which reinforced Sokolov’s concept of neuronal models. Nevertheless, there remained some criticisms of Sokolov’s theory including that Sokolov combined reflexive orienting with more voluntary aspects of attention (O’Gorman, 1973; Maltzman, 1979). Posner overcame some of the weaknesses in Sokolov’s theory with Posner’s three-part theory (Posner and Boies, 1971). I will discuss his work further under the section on approaches to measurement.

While Sokolov’s ideas followed Sechenov, Pavlov considered himself a strict follower of Sechenov (Ushakova, 1997). He called the orienting reflex the ‘What-is-it?’ reflex (Pavlov, 1927). He said that humans and animals would “immediately orientate their appropriate receptor organ” (e.g., ears for sounds, eyes for sights) to “make full investigation” of the change in the environment (p. 12). Behaviorism in the United States used Pavlov’s work to focus on observable behaviors and (early in behaviorism) expressed little interest in cognition. However, Pavlov had interest in cognition and the mind, as he expressed in his 1904 Nobel Lecture (Pavlov, 1967). He believed strongly in the ability of science to increase our understanding of behaviors such as reflexive orienting (Ushakova, 1997; Baars, 2003). He was, however, too confident in his ability to explain all human behavior through reflexes, and behaviorism tended to rely on his confidence (Baars, 2003).

### Behaviorists and Gestaltists

Some behaviorists also studied reflexive orienting; however, they tended to emphasize classical conditioning (Maltzman and Raskin, 1965). They did not often discuss mental processes such as attention (rejecting consciousness-centered psychologies). Nevertheless, aspects of the work on reflexive orienting must have been appealing because they discuss orienting as a precursor to learning. They also used orienting responsiveness to explain individual differences in discriminative ability (rather than referring to cognitive processes) and as a precursor to appropriate social behavior, for the same reason. Many behaviorists believed, as did Pavlov (1927) and Sechenov et al. (1863/1965), that cognitive processes were important to the individual but were not properly included in scientific explanations of behavior (Mandler, 2006). Indeed, Maltzman (1977) acknowledges that his “thinking about thinking” (p. 112) changed as he followed the findings in his experiments, which suggested some important cognitive aspects.

Maltzman et al. (1977) and Pendery and Maltzman (1977) investigated the orienting reflex using galvanic skin response as an outcome. The task was a semantic conditioning task similar to an oddball task with a critical word interspersed between filler words. When the critical word was presented, a tone sounded (instead of the usual noxious unconditioned stimulus) and the participant was expected to respond by either pressing or releasing a foot pedal. The data from those who were not informed of task contingencies (Pendery and Maltzman, 1977) or who were partially informed (Maltzman et al., 1977) were interpreted with larger galvanic skin response indicating greater orienting reflex. It is easy to see the cognitive processing in the “informed” aspect of the study. Several other research groups studied reflexive orienting with classical conditioning, including Badia and Defran (1970), Borton and Moore (1979), Raskin et al. (1969), and (more recently) Zimmer and Richter (2023). Typically, galvanic skin responses and cardiac responses (such as heart rate and vasomotor changes) are used as outcomes and cognitive processes are usually not discussed.

Even Gestalt theorists have a perspective on reflexive attention. For example, Gonen et al. (2014) found that observers pay attention to groups of objects when allocating reflexive attention, which is consistent with Gestalt perspectives. In figure-ground assignment studies, pre-cues only affect exogenous attention if they direct attention to an area within the figure-ground stimulus (Vecera et al., 2004). Nevertheless, some reports indicate that Gestalt theories do not explain some aspects of reflexive attention (e.g., see Jones et al., 2002). In addition, Gestalt explanations may not apply as widely as information processing approaches, and thus the latter may handle examination better.

### Information processing models

Today, reflexive attention is most commonly thought of in terms of information processing approaches, but there is historical nuance here as well. Müller’s influence (mentioned at the beginning of this section) undoubtedly extended to his advisee, Wilhelm Wundt. Like Müller (who wrote about the need to choose between “simultaneous sensations,” Müller, 1837, p. 622), Wundt wrote about the selective aspects of attention, which he called apprehension and apperception. The former indicated general awareness and the latter indicated “the focus of attention” (Wundt and Pintner, 1912/1973, p. 35). Wundt also wrote that unattended elements “disappear” from awareness (p. 36). His interest in psychology combined with his training in physiology suggests the close relationship between reflexes and information processing. Both fields can claim Wundt, although his recognition as the founder of the first psychology lab and his affiliation for experimental procedures involving reaction time (Robinson, 2001) led me to include him with information processing models.

Information processing models describe the processes of the mind in similar terms used for computers, such as storage, encoding, and retrieval. The “processing” referred to is usually measured by the speed and accuracy with which stimuli are handled cognitively. Speed is more precisely called response time (RT), which indicates the time from the onset of the stimulus to the completion of the response (such as a key press). A similar term, “reaction time” does not include the duration of the response, as it extends only from the onset or the stimulus to the initiation of responding. Accuracy is often the percentage of correct responses. Both RT and percent correct are often used as outcomes in cognitive research.

Information processing draws from the work of several Russian scientists mentioned earlier. For example, in event-related potential studies, Sokolov (1975) called a component of electrical activity in the cerebral cortex that arises in response to a novel stimulus appearing in a sequence of standard stimuli “mismatch negativity.” Note that Friedman et al. (2001) refer to the orienting response as mismatch negativity plus the novelty response, the novelty response occurring approximately 300 msec after stimulus presentation. The novelty response is associated with evaluating stimuli to determine the need for possible action. However, Sokolov (1960) emphasized the importance of the mismatch between a neuronal model and the current stimulus (his comparator model). In essence, this is an information-processing approach because it relates neural responses to cognitive responses that can be measured using reaction time (i.e., latency in ERP studies) and the strength of the response (i.e., amplitude in ERP studies). Sokolov (1960) indicated that the changes in the environment that triggered orienting (reflexive attention) could be “any increase, decrease, or qualitative change of a stimulus” (p. 189). However, he did not explain reflexive orienting that occurs following a familiar but task-relevant stimulus. More modern researchers have concluded that mismatch negativity is not the orienting of attention itself, but reorienting routinely follows mismatch negativity after approximately 200 more msecs (Fitzgerald and Todd, 2020). As Sokolov indicated, it is generally accepted that reflexive attention is the orienting of the brain’s resources to a novel object or location due to a change in the external environment (Sokolov, 1966; Donchin et al., 1984; Friedman et al., 2001; Zhu and Suzuki, 2017; Marois et al., 2018).

One technique within information processing began earlier in the nineteenth century when Donders (1869/1969) expounded the subtraction method, which is currently used in an impressive variety of studies, including for spatial subtraction between brain regions involved in a task and those involved in a baseline condition in neuroimaging studies. Donders method assumes that it takes brain resources to pass information from a sense organ to the brain and from the brain to make a response and, critically, that it will take extra brain resources (more time [temporal measurement] or more brain regions [spatial measurement]) if an additional process in the brain needs to be completed. Thus, the subtraction method is useful for temporal subtraction.

In the temporal subtraction method, differences in information processing are based on the latency to respond to a baseline condition (e.g., a task) compared to latency to a condition of interest. Helmholtz, a pioneer in experimental studies, contributed to the confidence that this approach would work when he measured the speed of conduction of a nerve impulse at about 30 meters per second (Hoff and Geddes, 1960). In addition, Helmholtz was the first researcher (ca. 1860) to demonstrate “covert” attention experimentally (Helmholtz, 1896/1924). Helmholtz would gaze into a wooden box through two pinholes and attend to a region of his visual field without moving his eyes in that direction. When a light briefly illuminated the box, he reported being aware of only the objects in the region he had been attending to (Carrasco, 2011). A century later, the work of Hick (1952) and Hyman (1953) provided support for the idea that RT was linearly related to the amount of information (e.g., the number of items to be processed or steps) required by a task. Generally, using the subtraction method temporally or spatially has allowed researchers to see inside the “black box” of the brain more precisely than could be done previously. RT methods continue to be critical to information processing research.

## Approaches to measuring of reflexive attention

### Reflexive attention in adults and children

As described above, the information processing approach is a way of conceptualizing cognitive processes such as attention using a model with input, output, and processing speed. It can be tested using computer tasks. In a task using Posner’s (1980) peripheral cueing paradigm, there is usually a central fixation cross, briefly appearing peripheral stimuli on the left, right, or in both positions, and then a target stimulus that appears on either the left or right. The cue is displayed for less time than is required to make an eye-movement (less than 70 msec) to simplify interpretation of task results. Such tasks are referred to as covert because they do not require head or eye movements (Posner and Rothbart, 1998). Cues are not responded to but, when present, usually draw attention so that participants’ subsequent responses to targets are slower if they follow contralateral (“invalid”) cues and faster following ipsilateral (“valid”) cues. The latter process is called facilitation.

Since cues provide information from both a location to attend to and instantaneous awareness of a cue, researchers often want to separate these processes. Fernandez-Duque and Posner (1997) explore evidence for the separation of these two processes and how they can be measured in Posner cueing tasks. The two processes are referred to as alerting (the instantaneous awareness) and orienting (to the location of the cue; see Posner, 1978; Posner and Raichle, 1994; Fernandez-Duque and Posner, 1997). One way to calculate alerting is to use neutral cues occurring bilaterally to the central fixation point and subtract RTs to targets following valid cues from the RTs following neutral cues.

Peripheral presentation of targets measures reflexive attention because participants need to reorient their attention from a central fixation point to respond to targets. If the cue were presented at central fixation (where attention already resides), we could not measure the process of attention being captured. When cues have a chance relationship with targets (e.g., 50% valid cues prior to presentation of the target), we know that the cues were attended to reflexively because no strategy can provide an explanation for faster responding following valid cues compared to invalid cues. There are increased RT costs associated with the extra steps of disengaging and moving attention to the location of a contralateral target. These steps are not involved when a cue and a subsequent target appear in the same location. That is, an invalid cue involves a disengage step prior to responding to the target that a valid (ipsilateral) cue does not require.

**Figure 1:**
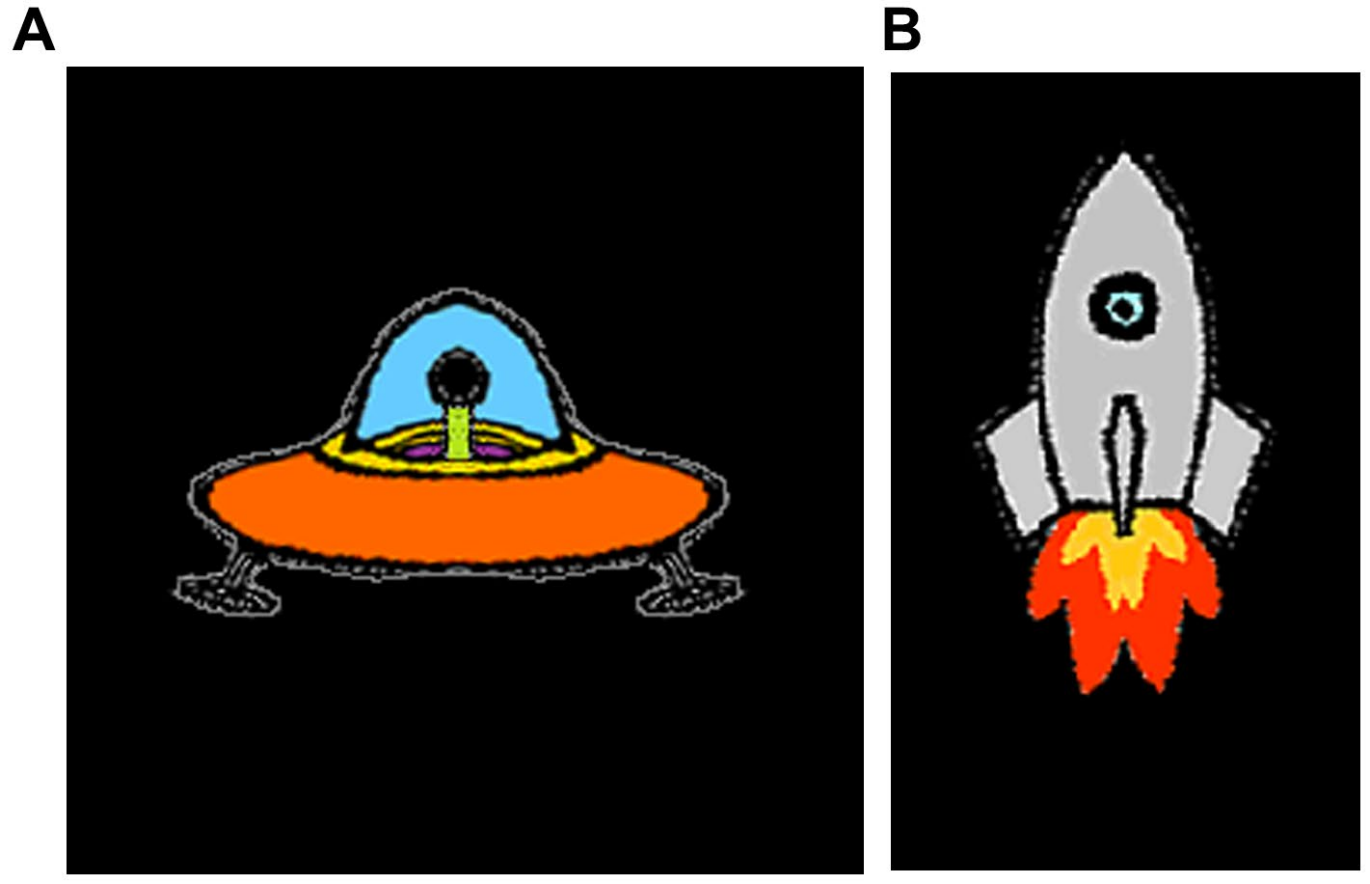
**(A)** The alien spaceship used in the child spatial cueing task and **(B)** the earth rocket used in the child spatial cueing task.

**Figure 2:**
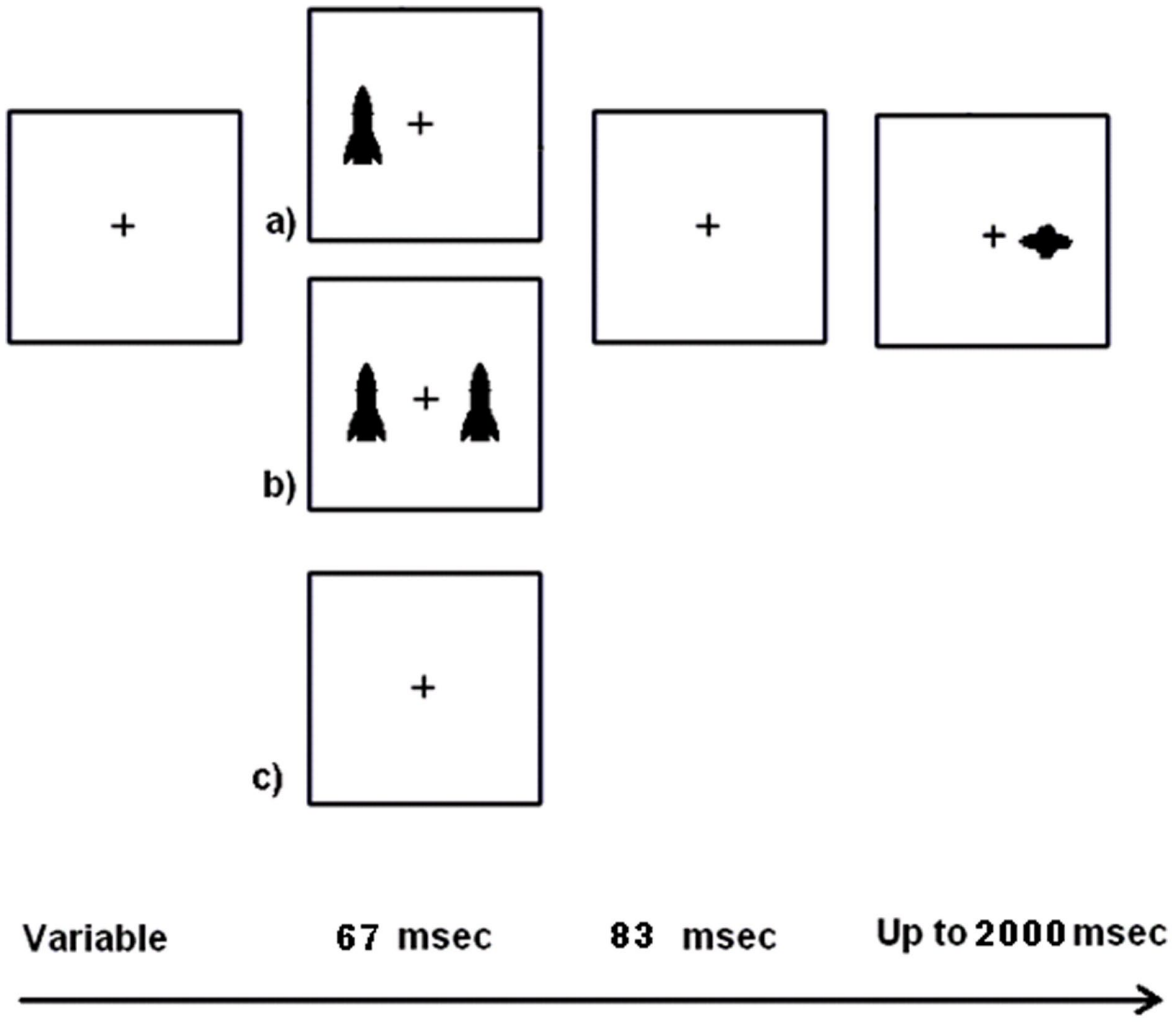
An illustration of a child version of a spatial cueing task.

Children can perform a modification of Posner’s peripherally cued orienting (reflexive attention) paradigm. In my research with children, I use cartoon alien spaceships and rockets as targets and cues, respectively and have a back story to create interest (see Figures 1A,B, 2; Lundwall and Hodges, 2018). The backstory involves the earth being under attack by aliens and children are instructed to hit the alien ships without hitting the friendly earth rockets. The task has been successfully used with children as young as 7 years old. While others have used the original spatial cueing tasks with children (Aman et al., 1998; Greenaway and Plaisted, 2005; Facoetti et al., 2006; Pergamin-Hight et al., 2016; Ronconi et al., 2018), these tasks have not been specifically designed to be child friendly and (probably consequently) are rarely used with children younger than 11 years old.

Several other researchers have used a modified peripherally cued orienting paradigm with children. Enns and Brodeur (1989) found that children (ages six or eight years) processed targets in non-cued locations more slowly than adults (approximately 20 years of age), thus providing evidence of the development of reflexive attention across this age range. Schul (2003) likewise found that orienting and disengaging attention were progressively more accurate and faster through the school years (7–17-year-olds). As a final example, Liu et al. (2017) found the peripheral cueing effect was intact in both poor and typical readers in a Chinese sample of second and third graders who were approximately 8 years old at the first timepoint.

Despite the popularity of the Posner paradigm, there are other reflexive attention tasks that are useful with children. Flanker tasks have distracting cues on either side of a central cue that can reflexively draw attention (Eriksen, 1995). Rueda et al. (2004) created a child-friendly flanker task using fish with their noses pointing left or right. For a review of findings using the child version of the flanker task, see Mullane et al. (2016).

Additional reflexive attention tasks for children include spatial cueing tasks involving social cues such as gaze direction (Kylliäinen and Hietanen, 2004; Marotta et al., 2014). However, gaze following is not strictly reflexive as it requires experience in social situations early in life (Zohary et al., 2022). Search tasks and visually-guided cueing tasks are similarly not reflexive because they involve goals. However, I acknowledge that reflexive and effortful attention often interact (Ristic and Kingstone, 2009; Bourgeois et al., 2017; Smith and Grabowecky, 2020; Vilanova-Goldstein et al., 2022). Rose et al. (2019) have used a gap-overlap task, which is described under *Measuring Reflexive Attention in Infancy*.

### Indices and mechanisms of reflexive attention

There are some aspects of reflexive attention that serve as indices across the lifespan. Inhibition of return (IOR) is an automatic aspect of attention. It refers to limits on temporal information processing (Klein, 2000; MacPherson et al., 2003), and Posner and Cohen (1980) proposed IOR as the phenomenon that prevents attention from returning to a stimulus location or object once it has been processed by the brain. While a valid cue usually facilitates faster responding to targets, at longer delays between cue onset and target onset, a valid cue will increase RTs. Klein (2000) indicates that the facilitation typically changes to inhibition about 250 msec after cue onset. However, he and his colleagues indicate that the term “IOR” is overused as inhibition does not always indicate IOR and recommend the term “inhibitory cueing effect” for situations in which the cue is spatially uninformative (Klein, 2000; Hilchey et al., 2014; Redden et al., 2021). Research investigating IOR generally finds that effects depend on a combination of cue duration, the gap between cue onset and target onset, the spatial distance between cues and targets, and age (McAuliffe and Pratt, 2005; Lundwall et al., 2018).

Colombo (1995) discusses the mechanisms of reflexive attention, which he calls spatial orienting. These include engagement, disengagement, and shifts of attention, which are functions that represent attention being captured, released, and moved to a new location. Additional indices of reflexive attention include facilitation and inhibition, which decrease and increase response times, respectively, depending on cue to target timings. He views fixation duration as indicative of the ability to disengage attention to allow it to be moved from one stimulus location (e.g., where a cue has appeared) to another (e.g., where a target has appeared). Difficulty disengaging attention could also be considered a failure to inhibit the current location of attention to engage another location. These ideas will be discussed further in the next section.

The effects described above apply primarily to measuring the reflexive attention of adults and children in middle or older childhood. To measure the development of reflexive attention more thoroughly, we need to be able to measure it in infants. The point of early identification is early intervention (Bradshaw et al., 2015). While attention training may not be possible (Wass et al., 2011; Peng and Miller, 2016), intervention in other ways (providing food security, additional academic support) are possible and may require early identification (Reid et al., 1999; Diamond et al., 2007; Blair and Raver, 2014).

## Measuring reflexive attention in infancy

### Tasks used to study infant reflexive attention

Reflexive attention is a relatively unique form of attention in that it can be measured from infancy to old age. As with much of development, we expect to see reflexive attentional improvements with age (Gibson, 1969; Hagen and Hale, 1973; Pick et al., 1975; Levy, 1980). Infants appear to rely more on automatic orienting than children or adults, who rely more on volitional orienting (Rothbart et al., 2011). It makes sense for infants to rely on suddenly appearing or moving stimuli as an indication of relevance. In a world of many unknowns, attending to suddenly appearing or moving stimuli reduces the number of surprises and makes the world a little more predictable (Kouider et al., 2015; Smith et al., 2018). The improvements often follow a cascading pattern with later developed skills tumbling into existence depending on the earlier developed skills (Oakes and Rakison, 2020, pp. 102–122). For example, Levy (1980) attributes improvement in reflexive attention tasks to more efficient strategies to deploy attention. Although this sounds effortful, strategy can impact reflexive attention because several processes of attention work together (Ristic and Kingstone, 2009). Both the rapid accrual of experience and an innate unfolding of brain development occur as we age and interact over our lives.

However, infants are not capable of understanding and following instructions and have limited motor control, making impossible their completion of key presses or most of the tasks we use to gather information from older children and adults. Therefore, measuring cognitive processes in infancy takes creativity. Several infant researchers have shown such creativity by using latency to first look (a form of looking preference) to infer processing time. For example, Cohen's (1972) and Cohen et al.’s (1975) work focused on the difference between attention-getting and attention-holding using a looking preference paradigm. To better communicate what we mean by looking preference, Rose et al. (1982, 1988) along with Hunter and Ames (1988) explored explanations for this observed behavior. In general, they found that infants return to a familiar stimulus when they have not finished processing it and a novel stimulus when they have finished processing the familiar stimulus. Since more difficult stimuli take longer to process, more difficult stimuli and younger infants often show a familiarity preference. Hunter and Ames (1988) made this particularly clear in their illustration of four differently aged infants being tested at two time points. Whether a researcher will see familiarity or novelty preference in these infants depends on which time points researchers examine. Essentially, Hunter and Ames (1988) say that the shape of the familiarity-novelty preference curve does not change, but the harder the task is for an infant (which partially depends on their age), the longer it will take to complete the familiarity-preference, novelty preference cycle and the more likely a familiarity preference will be observed at both time points.

Information processing speed is one possible explanation for seeing familiarity preference instead of novelty preference. There is another possibility, however. Colombo (1995) has indicated that looking preference could mean difficulty disengaging attention as well as information processing speed. Difficulties with disengagement have been noted by Posner’s group. For example, Johnson et al. (1991) found that only older (4-month-old) infants could disengage attention. Fischer et al.’s (1984) task, described below, is specifically designed to assess the ability to disengage attention.

Fantz (1961) used infant preferential looking to study aspects of infant perception. He used a method that pairs different stimuli (e.g., stripes, bull’s eye, checkerboard, square, and circle) and compared the looking times to each stimulus in a pair (Fantz, 1965). Fantz postulated that when infants look longer at stimuli it means they are still processing information from the stimulus (Fantz and Fagan, 1975). Researchers using his paradigm typically find that infants prefer looking at some stimuli (e.g., faces, high-contrast visual patterns, motion). There is no clear correct response, but this task demonstrates selective attention, even at birth (Johnson et al., 1991).

Fischer et al. (1984) developed the gap-overlap task (aka gap paradigm). Because the gap-overlap task assesses disengagement of attention using central and peripheral stimuli, it can be classified as an exogenous task (and Fischer’s “express” saccades are another name for reflexive orienting). Disengagement is not necessary in the gap conditions because the central stimulus disappears but is necessary in the overlap condition. Hood and Atkinson (1993) studied infants using this task and found that all age groups from 1.5-to 6-months-old took significantly longer to refixate in the overlap condition compared to the gap condition. However, there was also a significant interaction that demonstrated that the effect of overlap was greater for the younger age group. The shorter times to make saccades by older (3-and 6-month-old) infants compared to 1.5-month-old infants supports cortical disengagement explaining differences in reflexive orienting. Additional researchers have used peripheral stimuli to test for disengagement from central stimuli are consistent with the idea that older infants disengage attention more reliably than younger infants (Johnson et al., 1991).

**Figure 3:**
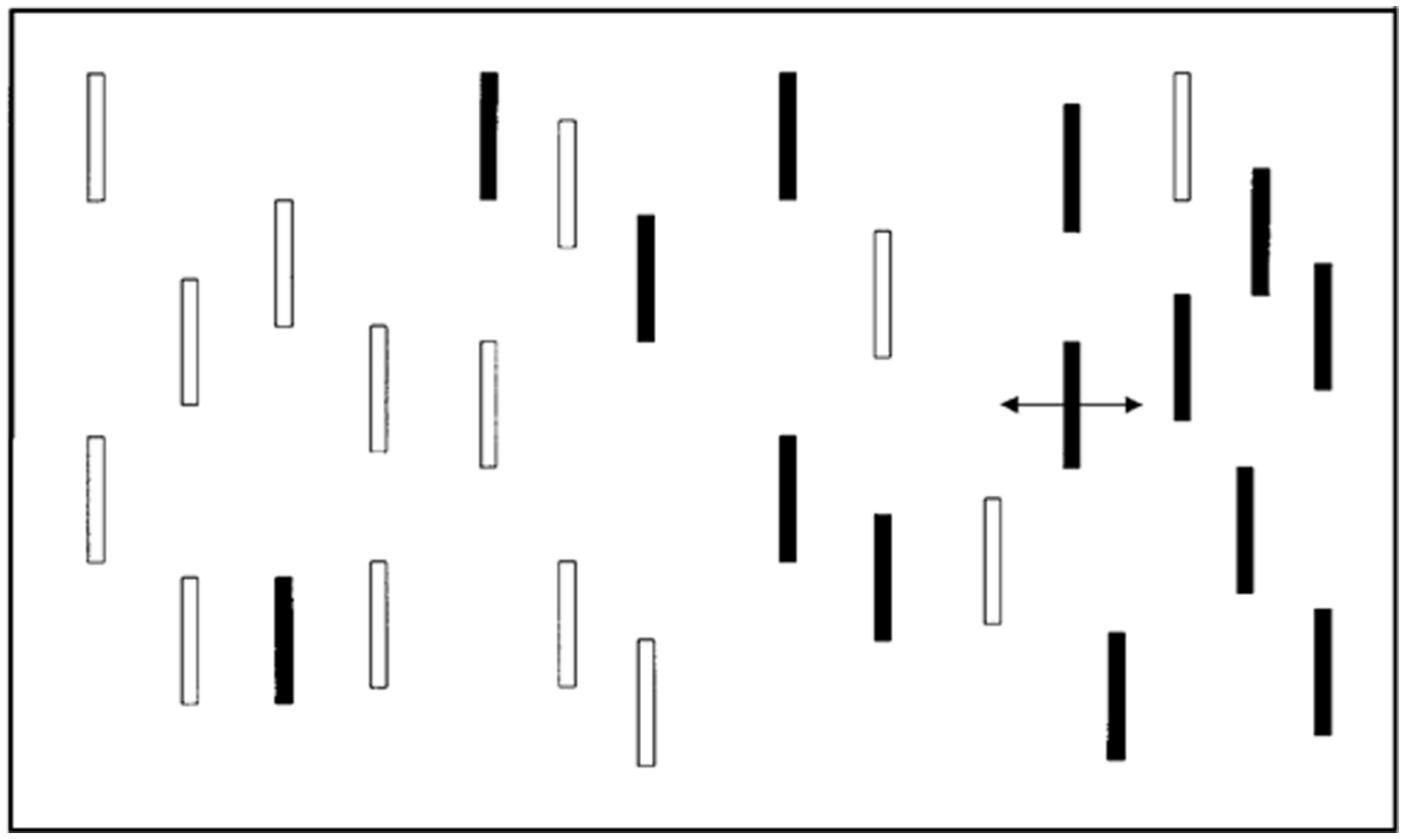
Representation of infant visual display for “moving bar” task. The actual presentation uses a medium gray background with lighter gray and darker gray bars.

Other reflexive attention tasks also rely on preferential looking. Harman et al. (1994) used a central attractor display followed by a unilateral left or right peripheral stimulus (see Figure 3). The attractor display then appeared again followed by bilateral peripheral stimuli. Researchers are able to measure the tendency to switch locations or perseverate at the location at which the unilateral stimulus appeared. They used the tendency to switch (rather than perseverate) as a measure of inhibition of return. Inhibition of return refers to a reduction in the processing of a stimulus that has been recently processed and indicates that the brain has already processed that stimulus (Oakes and Rakison, 2020, pp. 22–32; Clohessy et al., 1991; Pratt and Abrams, 1995). The task can also be considered a novelty preference task because infants tend to look to the side of the display where there was previously no target.

In another example of creativity in studying infant reflexive attention, Johnson et al. (1994) trained 4-mo-olds in an anti-saccade task. Although the anti-saccade portion of the task can be considered endogenous, this study strengthened evidence that infants can covertly attend to cues because they were faster to respond to targets in the occasional ipsilateral location from the cue. The study findings also argue against the idea that the cue is only impacting the eye movement system.

To work with infants, Teller (1997) combined preferential looking with a forced choice procedure. She presented infants with a sine wave grating on one side of a display and, on the other side, a gray patch matching the overall intensity of the grating. Based on the infant’s looking behaviors, the adult who is observing the infant (but not the display) makes a forced choice about the right versus left location of the grating. Teller argued that, because she had a clear external reference point of what was correct (the side with the grating can only be seen above a certain acuity level), the task was an improvement over other preferential looking tasks with no clear “correct” response.

**Figure 4:**
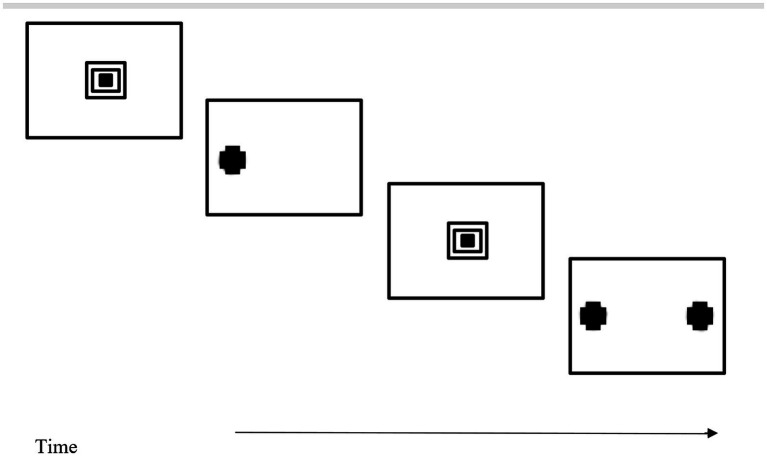
Representation of the “location novelty” task. Frames one and three represent attractor stimuli that appear to loom towards the infant. RT and percent correct can be obtained from frames two and four. Frame 2 measures the infant’s tendency to orient to a new location. Frame 4 measures the infant’s tendency to perseverate vs. switch locations that have already been attended (i.e., in frame 2) and thus assesses perseveration. Adapted from Harman et al. (1994).

Often, researchers will take the correct response to be what an adult would do. Dannemiller and Nagata (1995) modified Teller’s forced-choice preferential looking task to investigate the development of reflexive visual orienting to movement. The two sides of Dannemiller’s display had clearly visible vertical bars but, on one side of the display, one of the vertical bars oscillated horizontally. The rest of the bars served as distractors (see Figure 4). Movement is usually difficult for adults to ignore (i.e., movement captures attention reflexively). However, there are developmental changes across infancy (Dannemiller and Stephens, 2000) such that movement only gradually becomes compelling for infants.

Dannemiller and Nagata’s (1995) task is an attention task because infants “select” (attend to) the side with the moving bar at significantly above chance. Nevertheless, the task is different from visual search because infants, having limited understanding of language, cannot follow instructions. The percentage of “correct” judgments can be used as a simple measure of the selectivity of orienting. Percent correct refers to the percentage of instances where the observer’s decision as to the side of the display where the infant was looking matched the side of the display where the moving bar was located. Additionally, the latency for the observer to make these judgments is recorded, and this average latency can be used as a dependent measure.

Ross-Sheehy et al.’s (2015) group developed the Infant Orienting With Attention (IOWA) task. The IOWA task involves valid, invalid, double, tone, and no cue conditions. The tone and no cue conditions serve as baseline. As is typical, RTs were decreased in the conditions that contained a valid spatial cue (valid and double cue conditions) compared to the invalid cue condition. RTs for baseline conditions fell in between those for valid and invalid conditions. Younger infants (5 months) were also slower to respond than older infants (10 months). Interestingly, the effectiveness of the pre-cue improved with older infants (an invalid cue led to more incorrect responses for 7-and 10-month-olds). In a later study, Ross-Sheehy et al. (2017) found that pre-term infants experience altered postnatal developmental trajectories with 5-month-old preterm infants experiencing significant deficits in orienting speed and error rate compared to full-term 5-month-old infants. Ten-month-old preterm infants had relatively less deficit than 5-month-old infants compared to full-term 10-month-old infants. They interpret their findings as indicating the critical role that postnatal visual experience imparts and suggest checking infant visual orienting may be a sensitive measure for assessing attentional delay.

### Findings for infant reflexive attention studies

Research psychologists have learned many things about how infants think from the looking preference paradigm. We have learned that infants will also turn their eyes and head to orient to stimuli in the periphery from birth (Goren et al., 1975) and covertly by about 3 months old (Richards, 1988). One of the most important things we have learned is that reflexive attention develops gradually (Richards, 1988; Johnson and Tucker, 1996; Dannemiller, 2005; Abundis-Gutiérrez et al., 2014), although voluntary attention develops even more gradually (Colombo, 2001; Ristic and Kingstone, 2009; Fisher et al., 2013).

Studying developmental patterns often involves comparing children of different ages in cross-sectional research. For example, Colombo (1995) notes that infants who look longer at a fixation stimulus tend not to disengage or shift visual attention as easily. In a series of studies using global–local stimuli, Colombo and colleagues conclude that because the duration of visual fixation is correlated with information processing speed, longer-looking infants tend to process local properties of a stimulus earlier than shorter-looking infants, who process global properties first (Colombo et al., 1991, 1995; Frick et al., 2000).

We can also discover developmental patterns by correlating differences found in one age with those found at another age. In longitudinal studies, if researchers find lagged reliability (a.k.a. stability) of scores within individuals from infancy to childhood, this suggests infant attentional tasks may be tapping cognitive abilities that are foundational for later child attention and academic achievement. Stability refers to consistency in the relative rank ordering of individuals with respect to the expression of ability (Bornstein et al., 1997). As an example of poor stability, infant IQ measures do not correlate well with childhood IQ measures. This implies that these tests are not measuring the same construct, probably because infant IQ measures are based on sensorimotor tasks and cannot be expected to predict accurately later cognitive functioning (Fagan and Singer, 1983). This finding led researchers to instead examine cognitive processes that might underlie both infant and child performance. When such stability is evident between infant and child measures then it tells us something about what is being measured in infancy. In this effort, studying information processing has been a productive approach.

As one example of lagged reliability, preferential looking tasks can be used to predict childhood intelligence scores (Facoetti et al., 2000). Fagan estimates concurrent reliability for his task at *r* = 0.60 (Fagan and Singer, 1983). Lagged reliability from infants (4 to 7 months old) to 4-and 7-year-olds was r = 0.37 and 0.57, respectively (Fagan and Singer, 1983). Although, in this case, Fagan’s concurrent and lagged reliability estimates to 7-year-olds are just below concurrent reliability, Colombo (1993) suggests that stability is often significantly better than concurrent reliability, possibly due to inconsistency in infant behavior (e.g., fussiness or lack of interest in the task). As Colombo points out, however, if the child task is highly reliable, then a moderately low reliability on the infant task can still provide reliable lagged predictions. For example, if the infant measure’s reliability is 0.40 and the child measure’s reliability is 0.90, then the square root of the product of 0.40 and 0.90 is 0.60, which would be the reliability of the lagged prediction. Changes in facilitation and inhibition over development likely occur precisely because the brain is not finished developing at birth.

In further evidence, infant time to habituate predicts child intelligence (IQ; Fagan and Singer, 1983). This implies that complex intellectual functions necessary for academic achievement in childhood may be traced to the integration of simpler functions (Colombo, 1993) and likely occurs because preferential looking indicates time spent processing information (Cohen et al., 1971; Fantz and Fagan, 1975; Fagan and Singer, 1983; O'Connor et al., 1984; Colombo and Mitchell, 1990; Rose and Feldman, 1990). Individuals show stability across their own development. Correlations between infant cognitive tasks and child IQ range from *r* = −0.38 to −0.75 (Colombo and Mitchell, 1990; Bornstein et al., 1997).

Among the many things learned studying reflexive attention is that reflexive saccades to peripheral targets are likely present from birth (Richards and Hunter, 1998), but infants take more steps to precisely reach a target (Aslin and Salapatek, 1975; Salapatek et al., 1980). Inhibition of return, on the other hand, likely develops between 3-and 4-months of age (Clohessy et al., 1991; Hood and Atkinson, 1993; Harman et al., 1994; Johnson and Tucker, 1996; Richards, 2000; Richards, 2001) although it is sometimes document in the first days of life (Valenza et al., 1994; Simion et al., 1995). Cues can also facilitate eye movements and this ability to benefit from cues (facilitation) develops between the first 3-and 7-months of life (Johnson and Tucker, 1996; Hood et al., 1998; Richards, 2000). Discrepant features or moving stimuli can also attract infant attention reflexively at young as about 2-months old (Salapatek, 1975; Dannemiller and Freedland, 1993).

Infants as young as 3 month-old can complete reflexive attention tasks that are similar to tasks completed by older children and adults, such as the spatial cueing task used by Markant et al. (2014). Johnson and Tucker (1996) used suddenly appearing stimuli in a manner similar to Posner (1980). Richards (1988) used infant-initiated looking paradigms. Markant and Amso (2013) used a spatial cueing task that included cues, targets, and foils. Foils looked dissimilar from but appeared when targets would normally appear on the opposite side expected (contralateral to cue in the facilitation condition and ipsilateral in the inhibition condition). Infants were randomly assigned to the facilitation or IOR conditions. In the facilitation condition, targets appeared in the cued location and foils appeared in the non-cued location. In the IOR condition, the targets appeared in the non-cued location and the foils appeared in the cued location. Infants were given a preferential looking memory test (“correct” was defined as novelty preference). Markant and Amso found that inhibition conditions improved memory compared to facilitation conditions in 9-month-olds who had improved memory following inhibition trials. In a follow-up study, Markant et al. (2015) found fMRI results that indicate the memory improvement was driven by activity in the visual cortex because better suppression of the previously attended target during encoding predicted subsequent improved recognition memory.

## Conclusion

Part of the rationale of conducting tests of early infant cognitive skills and associating these tests with later outcomes, such as academic achievement, is to understand the development of cognitive processes. Reduced reflexive responses to peripheral stimuli might indicate risk for future difficulties in school or attention difficulties in daily life.

In a similar manner, reflexive attention tasks might help determine if an infant has or is likely to develop an attentional deficit, which, in turn, will help guide intervention. This will be especially helpful if identification occurs early so intervention can begin before brain development is complete.

Reflexive attention is a useful predictor of later aspects of cognitive skill and functioning. In part, this is supported by evidence that infants who process stimuli quickly tend to have shorter latency to first look in infancy and higher IQs in childhood. Although not voluntary, reflexive orienting is important to learning and memory because it triggers attention to new stimuli and allows further processing, memory, and learning to commence. If we did not have this propensity to notice novel events, we would fail to learn anything new from our environments. Infant looking behaviors support the idea that reflexive orienting is an important way infants learn about the world, and likely remains important for older children and adults.

## Author contributions

The author confirms being the sole contributor of this work and has approved it for publication.

## Conflict of interest

The author declares that the research was conducted in the absence of any commercial or financial relationships that could be construed as a potential conflict of interest.

## Publisher’s note

All claims expressed in this article are solely those of the authors and do not necessarily represent those of their affiliated organizations, or those of the publisher, the editors and the reviewers. Any product that may be evaluated in this article, or claim that may be made by its manufacturer, is not guaranteed or endorsed by the publisher.
